# Maize Detection and Row Extraction Using Maize–YOLO and IPM–Clustering Method for Autonomous Agricultural Navigation

**DOI:** 10.3390/s26102952

**Published:** 2026-05-08

**Authors:** Tao Sun, Junzhe Qu, Chen Cai, Yongkui Jin, Songchao Zhang, Feixiang Le, Xinyu Xue, Longfei Cui

**Affiliations:** 1 Nanjing Institute of Agricultural Mechanization, Ministry of Agriculture and Rural Affairs, Nanjing 210014, China; suntao@caas.cn (T.S.); qujunzhe1213@163.com (J.Q.); caichen@caas.cn (C.C.); jinyongkui@caas.cn (Y.J.); zhangsongchao@caas.cn (S.Z.); lefeixiang@caas.cn (F.L.); 2 Sino-USA Pesticide Application Technology Cooperative Laboratory, Nanjing 210014, China

**Keywords:** maize detection, crop row extraction, Inverse Perspective Mapping (IPM), autonomous agricultural navigation

## Abstract

Real-time and accurate crop row extraction is a fundamental requirement for vision-based perception in autonomous agricultural machinery. In maize fields, however, row detection is easily affected by variable illumination, leaf occlusion, weed interference, and uneven soil backgrounds, which can reduce the reliability of both GNSS- and image-based navigation methods. To address these challenges, this study proposes a plant-oriented crop row perception framework that reconstructs row structures from individual maize plant detections. A lightweight detection model, named Maize–YOLO, was developed based on YOLOv11n for maize seedling detection. Three key improvements were introduced to enhance the balance between accuracy and efficiency. First, the C3k2_Faster_CGLU module replaces the original C3k2 block to reduce redundant convolutional computation while improving selective feature representation through convolutional gated linear units, thereby enhancing robustness under complex field backgrounds. Second, a lightweight shared detection head, Detect_LSH, was designed to share convolutional parameters across multi-scale feature maps and adaptively adjust feature amplitudes, reducing detection-head redundancy while maintaining multi-scale prediction capability. Third, a Layer-Adaptive Magnitude-Based Pruning strategy was applied to remove low-contribution channels and further improve computational efficiency for CPU-based deployment. Experimental results on field-collected maize seedling images showed that Maize–YOLO achieved an mAP@0.5 of 97.6%, reduced GFLOPs by 61.9%, and maintained a CPU inference speed of 84.4 FPS. After plant detection, row centerlines were estimated using an IPM–DBSCAN–LSM pipeline, which transformed detected plant centers into a quasi-top-view space, clustered them into crop rows, and fitted continuous centerlines. The extracted crop rows reached a positional accuracy of 98.6%, with a mean angular deviation of 0.44°. These results demonstrate that the proposed method can provide accurate, lightweight, and real-time crop row perception for autonomous agricultural navigation and precision field operations.

## 1. Introduction

Intelligent development has become the driver of traditional farming automation and informatization in recent years. Agricultural intelligent machinery has become a practical solution to enhance field production, resource-use efficiency and environmental friendliness. Maize, as a major global cereal crop, is vital to food security, making its precision management an essential area of research. High-precision autonomous navigation is a prerequisite for precision seeding, variable-rate fertilizing and efficient harvesting, which have been the main approaches of intelligent field operations. Since the introduction of autonomous navigation concepts in the 1980s [1,2], continuous advances in sensing, control, and machine vision technologies have driven remarkable progress. However, in dynamic outdoor conditions with uneven ground, illumination changes, and occlusion of crops, it is still difficult to guarantee both positional accuracy and real-time performance [3,4].

Currently, autonomous navigation in agricultural machinery is mainly developed in three technological fields: Global Navigation Satellite System (GNSS), LiDAR (Light Detection and Ranging), and machine vision. However, among these methods, GNSS-based navigation is still the dominant method to realize high-precision positioning in the open field, which can reach centimeter-level accuracy under good signal conditions. This feature contributes to improving the work accuracy and efficiency of agricultural machinery [5]. In a typical GNSS-based navigation procedure, a reference path is derived based on the field geometry and agronomic constraints. During the operation, the onboard GNSS receiver generates a stream of absolute position coordinates, which are then compared with the preplanned path to determine lateral deviations. The controller then issues steering corrections in real time to maintain the vehicle’s alignment with the target route [6]. Despite its maturity, this absolute-coordinate tracking strategy exhibits inherent limitations because it lacks direct perception of the crop row structure [7,8]. The system relies solely on predesigned trajectories, making it insensitive to spatial variations in actual crop row position. Two major consequences often arise: (1) crop damage risk—during post-sowing operations such as weeding or spraying, even small misalignments between the planned path and real crop rows can lead to plant trampling and subsequent yield loss and (2) limited environmental adaptability—terrain undulation, surface residues, or unstructured obstacles may change the vehicle’s pose, and GNSS-only guidance cannot dynamically maintain the optimal relative alignment between key operational components (e.g., sprayers, cutters) and their working targets (e.g., crops, weeds, soil) [9].

In addition to single-sensor navigation methods, GNSS–vision fusion has been increasingly investigated to improve the robustness of agricultural autonomous navigation. In such systems, GNSS or RTK-GNSS generally provides global positioning and field-level path constraints, whereas machine vision supplies local environmental perception, such as crop row detection, obstacle avoidance, and row end recognition [10]. This combination can compensate for the limited environmental perception of GNSS and the limited global planning capability of vision-only navigation. However, existing GNSS–vision fusion methods still face several limitations in practical field environments. First, GNSS accuracy may degrade because of signal blockage, multipath effects, satellite availability, and adverse weather conditions. Second, visual perception remains sensitive to illumination variation, shadows, weed interference, crop growth stages, and leaf occlusion [10]. Third, many fusion frameworks mainly improve vehicle localization or trajectory tracking, but they do not directly solve the problem of accurate and real-time crop row structure extraction from complex field images. Moreover, tightly coupled GNSS/IMU/vision systems often require additional sensors, calibration procedures, synchronization, and computational resources, which may increase deployment complexity and cost [11]. Therefore, even in GNSS–vision-integrated navigation systems, a lightweight and robust crop row perception module is still necessary to provide reliable local row geometry for real-time path guidance and precision field operations.

To overcome the constraint of GNSS in relative pose estimation between farm implements and crop rows, vision-based sensing and LiDAR for field perception and autonomous navigation have recently been more frequently adopted by investigators [12,13]. LiDAR is based on the time-of-flight (ToF) principle, where it calculates the distance to objects by sending out laser pulses and measuring the time between their emission and the reception of the reflected signals [14]. The resulting three-dimensional point cloud offers centimeter-level spatial resolution and captures essential structural information of the field environment, including crop row geometry, terrain variation, and potential obstacles [15]. Even though LiDAR has accurate spatial awareness, its use for farm machinery is still limited due to practical problems, such as the high cost of the equipment, complex calibration process and huge amount of data processing [16]. Therefore, the trade-off among sensing accuracy, computational complexity, and cost effectiveness has become a problem of interest in the context of large-scale implementations. By contrast, crop row detection based on machine vision is a more lightweight and cheaper solution. Due to the abundance of visual information and the ability to be easily incorporated with other systems, vision-based techniques have quickly become one of the most active and successful research fields in agricultural unmanned navigation.

By processing field images in real time, vision sensors are capable of providing a direct estimate of the lateral offset and heading misalignment of the agricultural machine with respect to the crop rows. In contrast to GNSS-based navigation, vision-based systems do not follow predetermined global trajectories, thus enhancing flexibility and environmental adaptability [17]. In addition to these advantages, vision-based solutions are more cost-effective, provide more information and are easier to integrate into systems, which has promoted vision-based navigation to a core issue in agricultural robotics research [18]. In such high-precision field operations as weeding and spraying, machine vision has gained importance as a sensing technique for crop row localization and weed discrimination [19,20,21,22]. Traditional image-processing and deep learning-based methods are the two major categories of vision-based crop row detection approaches. Classic pipelines of color thresholding and segmentation are mainstream for discriminating vegetation from soil background, which enables further crop row extraction [23,24]. For example, Zhai et al. [25] introduced binocular stereo vision for spatial reconstruction of crop rows with a success rate of 92.78%. Nevertheless, the algorithm suffered a high computational burden, and dense stereo matching consumed 634 ms on average. Bakker et al. [26] fused IPM and grayscale segmentation together with the Hough transform to obtain an estimate of the heading deviation angle, which successfully resolved the region-of-interest uncertainty in the case of non-strict parallelism of rows. But once again, the computational burden limited the time performance. Xu et al. [27] performed excess-green vegetation feature extraction and Otsu thresholding for maize–weed separation and seedling localization by combining morphological reconstruction with projection histograms; however, the approach was only promising when crop growth was relatively homogeneous. To conclude, traditional vision algorithms for detecting plant regions heavily rely on manually designed low-level features, such as color and texture, which have poor generalization under varying illumination, shadows, or vegetation coverage. Such constraints may cause unstable line fitting, low robustness, and limited real-time implementation in complex field environments. Therefore, the accuracy, robustness, and time complexity of vision-based crop row detection still deserve to be further improved in future work [28,29].

Improvements in deep learning have significantly promoted agricultural machine vision in recent years. Owing to their ability for end-to-end feature extraction, convolutional neural networks (CNNs) achieved superior detection performance, inference time and robustness, and were applied to various agricultural applications such as weed detection [30], pest and disease diagnosis [31], and autonomous navigation [30]. In the context of crop row detection, we find that current deep learning-based solutions can be categorized into two groups: segmentation-based and object-detection-based methods. In the scope of segmentation-driven solutions, Adhikari et al. [32] utilized the ES-Net to segment rice row images and then proposed a sliding-window-based method to cluster and fit crop lines in ROIs (Regions of Interest) for crop row detection. The navigation trajectory was then determined with the midline between two neighboring rows. Ponnambalam et al. [33] considered the uneven inter-row distances in strawberry plantations and proposed using a SegNet-based segmentation model with an adaptive ROI selection parser to handle stable navigation in different planting structures. Yang et al. [34] enhanced adaptability in the field by adopting a modified U-Net model to segment potato rows, with an edge feature extraction step based on alternate line scanning and K-means clustering, followed by least squares fitting to trace the row center lines, establishing robust navigation performance. It is also worth noting that object detection-based methods have also shown great promise. Shi et al. [35] presented the DCGA-YOLOv8 model, with deformable convolution (DCN) and a global attention mechanism, which attains the highest detection accuracy for cabbage, kohlrabi and rice. Through integration with the DBSCAN clustering algorithm, the method achieved navigation line reconstruction with centimeter-level precision in complex field environments. Gong et al. [36] put forward an enhanced YOLOX–Tiny model for detection of maize rows operating on bounding-box centers as control points and utilizing least squares fitting to calculate row centerlines. Their system attained an average heading error of just 0.59° under changing illumination, bringing a new level of navigation accuracy to automated intelligent weeding robots. Li et al. [37] tackled the problem of early-stage maize detection in the presence of strong illumination and weed challenge by proposing an enhanced YOLOv8-G model that detects individual seedling centers and conducts adaptive clustering via the Affinity Propagation technique. With a detection accuracy of 96.52%, the fitted centerline performs well in seedling-stage maize fields, and deep learning has made crop row detection more automated, robust, and accurate than traditional vision algorithms, enabling high precision, real-time operation, and stability.However, existing models remain computationally intensive, with a complex structure and unpleasing inference efficiency, which is not conducive to real-time application on embedded processor settings in agricultural machinery. Therefore, more effort should be devoted to lightweight network designs, efficient feature representation, and inference optimization, leading towards a more practical trade-off between detection accuracy and computational efficiency.

Studies show that classical vision-based methods cannot provide high-precision crop row detection under complex field environments, while deep learning-based approaches demonstrate better robustness and generalization in different environments. Object detection methods can efficiently localize each plant by employing bounding-box regression, but they lack explicit spatial continuity and topological structures that encode row information. As a consequence, post-processing methods such as DBSCAN clustering are usually needed in order to recover row centerlines, which results in further computational overhead and increases the sensitivity of the extracted lines to localization noise. On the other hand, methods based on segmentation can exploit denser spatial cues at the cost of greater computational complexity and are susceptible to misgrouping two nearby rows, which may hinder their application in a fast agricultural environment. In large-scale maize fields, visual scene complexity, shaped by illumination change, weed interference, various soil backgrounds and irregular seedling growing status, is likely to lead to either discontinuous or curved crop row patterns. These elements add to the difficulty of keeping the model accurate and robust when the environment changes. Therefore, a non-negligible trade-off always exists between detection accuracy and real-time performance, which greatly hinders the applicational reliability of autonomous navigation in farm machinery.

For better accuracy and efficiency, in this paper, an improved maize row detection model called Maize–YOLO based on YOLOv11 is proposed to address the aforementioned constraints. The method integrates a novel object detection backbone with a refined crop row modeling approach, which can effectively capture individual plant-level features while modeling global geometric continuity of row structures. By adopting structural improvements and a pruning-based lightweight design, Maize–YOLO can attain a good trade-off between detection accuracy and inference speed. The framework turns out to be a robust visual perception solution for real-time navigation and precise operations of unmanned agricultural machinery in complex field scenery due to its superior enhancements in robustness against illumination, occlusion and background variations.

## 2. Materials and Methods

**Figure 1:**
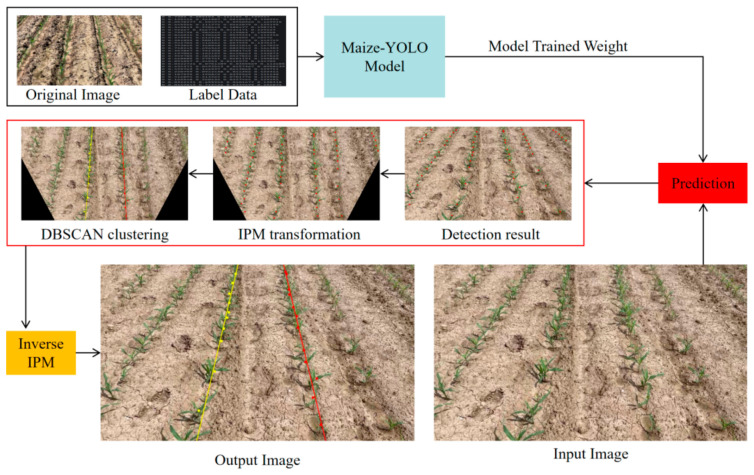
Flow chart of the crop row extraction.

The designed approach, named Maize–YOLO and IPM–Clustering, is based on an enhanced YOLOv11 framework to realize the high-precision detection of single maize plants and the exact reconstruction of the crop row configuration. As depicted in Figure 1, the entire architecture consists of two main stages: (1) crop plant detection—extraction of plant-level spatial information using an improved lightweight detector with detection of the centers of plants, and (2) crop row structure generation—a global row topology reconstruction from detected plant centers.

In the first stage, an improved YOLOv11n convolutional neural network is used for maize plant detection in field images, which supports accurate localization and identification of individual targets via bounding-box regression. In the second step, the center points of the detected boxes are used to perform dimension reduction and cluster analysis on the exposed spatial layout of the crop rows. Subsequently, the clustered results are regressed with the least squares method (LSM) to obtain continuous and smooth centerlines, which represent the geometric structure of the maize rows. Finally, the performance of the proposed method is evaluated based on detection accuracy and row-fitting error analysis across various field environments.

### 2.1. Image Acquisition and Dataset Construction

**Figure 2:**
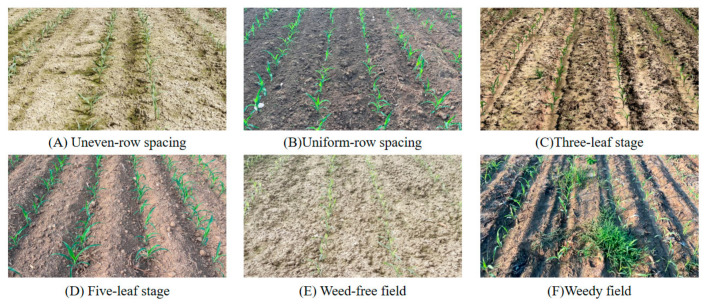
Illustration of dataset examples.

A field image dataset of maize seedlings was constructed for model training and testing. Image acquisition was conducted in three stages at three sites in eastern China. The first stage was conducted in Jiangzhuang Town, Gaomi City, Shandong Province, in June 2025; the second stage was conducted in Suining County, Xuzhou City, Jiangsu Province, in July 2025; and the third stage was conducted in Jiangning District, Nanjing City, Jiangsu Province, in July 2025. To improve sample diversity and model generalization, maize field images were captured under different weather conditions, background textures, and planting patterns (Figure 2). A total of 1555 images were obtained at different maize growth stages and under varying cultivation conditions. The images were captured using the rear camera of an iPhone 15 smartphone (Apple Inc., Cupertino, CA, USA) with an original resolution of 1080p (1920 × 1080 pixels). During field acquisition, the camera was held approximately 1.5 m above the ground and pointed toward the maize rows. To reduce variations in imaging geometry, the shooting height and viewing direction were kept as consistent as possible across different field sites. All images were saved in JPG format and resized to the network input size before model training, validation, and testing.

The LabelImg tool (Version 1.8.6) was used to manually annotate maize plants with rectangular bounding boxes, assigning all targets to a single category labeled “maize.” The annotation files were exported in YOLO format with the class of the object and its bounding box coordinates. The targets in the maize were tightly packed, with high partial occlusions, making object localization difficult [38]. After the annotations were done, the dataset was segmented into training, validation and testing subsets with an 8:2:1 ratio randomly, resulting in 1131 images for training, 283 images for validation and 141 images for testing. The scale, spatial, and visual diversities of the dataset can allow sufficient training of the model based on deep learning, which leads to the best enhancements in the YOLOv11 structures.

### 2.2. Object Detection Model

#### 2.2.1. YOLO Series Model

For agricultural applications, especially in crop row detection and autonomous navigation of field or farm vehicles, real-time performance is a decisive factor that directly affects the whole system’s performance. Although the fine-grained recognition results can be obtained using semantic segmentation algorithms, they are high in computational complexity, which requires a large amount of processing resources, and the time of inference is long. In addition, segmentation-based approaches typically necessitate post-processing to separate multiple crop row masks, which results in additional computational burden. Since most agricultural equipment uses onboard computing systems with limited hardware capabilities, the execution of such complicated networks typically leads to high latency. As a consequence, practical in-the-field performance of segmentation-based crop row extraction approaches is often hindered by real-time processing constraints.

The single-stage object detector YOLO (You Only Look Once) achieves exceptional inference speed and maintains competitive detection accuracy. Due to its trade-off between efficiency and accuracy, the models of YOLO have been widely applied in agricultural vision tasks such as crop detection, pest and disease surveillance, as well as field management [39]. In contrast to two-stage methods like Faster R-CNN, YOLO casts object detection as a single end-to-end regression problem, directly predicting bounding boxes and class probabilities from full images in one evaluation. This integrated framework enables drastic computational cost reduction and significant real-time detection performance improvement.

**Figure 3:**
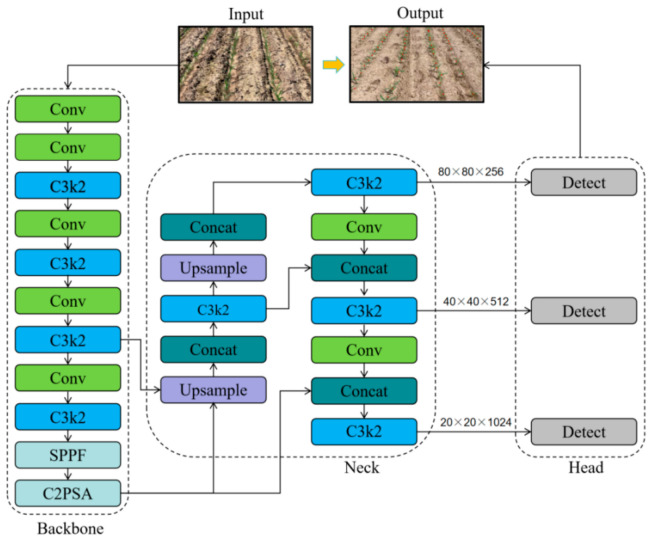
The original network structure of YOLOv11.

Since the release of YOLOv1 by Redmon et al. in 2015 [40], the YOLO series has undergone several generations of evolution in network architecture, feature representation, and inference optimization. Considering detection accuracy, model complexity, and deployment efficiency, YOLOv11n was selected as the baseline in this study. This selection was based on its lightweight architecture, good portability, and favorable balance between detection performance and computational cost. Compared with larger YOLO variants, the nano version is more suitable for real-time agricultural applications where onboard computing resources are limited. In addition, the modular network design of YOLOv11n facilitates the integration of the proposed C3k2_Faster_CGLU module, Detect_LSH head, and LAMP pruning strategy. To avoid arbitrary baseline selection, the suitability of YOLOv11n was further evaluated by comparing it with several representative lightweight detectors under the same experimental settings, as reported in Section 3.6. Therefore, YOLOv11n was adopted as the baseline framework for developing the proposed Maize–YOLO model. The framework of YOLOv11 is shown in Figure 3.

#### 2.2.2. Network Architecture for Maize–YOLO

##### C3k2_Faster_CGLU Module

In this work, the YOLOv11n model was used as the baseline network. In order to improve the feature extraction ability, we replace the original C3k2 module with a C3k2_Faster_CGLU module.

The C3k2 module is an important feature extraction module in the YOLOv11n backbone, which is based on the CSPNet (Cross Stage Partial Network) architecture [41]. It divides the input feature map into two parallel paths and recombines them at the Neck to learn multi-scale feature representation for better processing of spatial information. But the original C3k2 module uses several Bottleneck blocks in a stack, which causes channel waste and a high computational burden. In order to relieve the above problem, the Bottleneck units are replaced by FasterBlocks, named C3k2_Faster. This process simplifies the computations during the extraction of features and at the same time enhances the ability of the backbone to capture spatial information, thus offering a more sound basis for later maize target detection.

**Figure 4:**
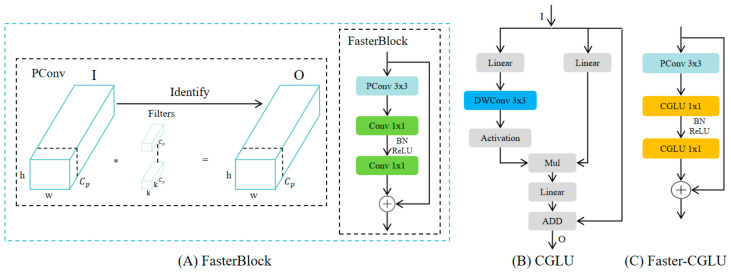
The improved C3k2 module structure diagram.

The FasterBlock, inspired by the FasterNet [42] design, is intended to reduce computational cost and memory usage by presenting efficient convolution layers. Its main novelty comes from the Partial Convolution (PConv) layer, where one-fourth of the input channels are convolved by the standard convolution, and the remaining three-fourths are directly skipped for the next fusion (Figure 4A). Compared to regular convolution, this pattern greatly reduces floating point operations (FLOPs) and memory accesses cost, with FLOPs of PConv being about one-sixteenth of that for standard convolution, while still providing effective feature propagation. The FLOPs and memory access of the PConv operation can be formulated as:

(1) $$P_{F}=h\times w\times k^{2}\times c_{p}$$

(2) $$P_{M}=h\times w\times 2c_{p}+k^{2}\times c_{p}^{2}\approx h\times w\times 2c_{p}$$

where h and w denote the height and width of the input feature map, respectively; k represents the convolution kernel size; and c_p_ denotes the number of channels involved in the convolution operation in PConv. In the Partial Convolution operation, only a subset of the input channels is processed by standard convolution, while the remaining channels are directly bypassed and later fused with the convolved features. Therefore, c_p_ is smaller than the total number of input channels, which helps reduce both FLOPs and memory access while maintaining effective feature propagation.

Although FasterBlock is more efficient, its receptive field is still mostly local, which limits its understanding of broader spatial relations. In maize seedling images (maize often overlaps and grows in dense clusters), relying only on local clues could desensitize the model to the overall crop row pattern geometry. To overcome this issue, a Convolutional Gated Linear Unit (CGLU) module [43] is integrated in the architecture. The CGLU is a combination of convolutional filtering and a gating mechanism, which can enhance informative responses and suppress noise-like activations, leading to more selective and flexible features. This modification enables the model to capture more global context information with insignificant additional computational overhead and is thus beneficial to the robustness and performance of dense object detection.

The CGLU is based on the conventional Gated Linear Unit (GLU) with a 3 × 3 depthwise convolution preceding activation in the gating branch. The only difference is that it makes the gating operator more locally spatially aware, but with negligible additional computations.

As illustrated in Figure 4B, the module has two branches: one branch consists of a depthwise convolution and a gating activation, which is computed to produce the spatially adaptive weights; the other generates the candidate feature map. They perform element-wise multiplication on their outputs and then linearly add the product back to the input feature to obtain the final output. Thus, local information participates in gating, which enables the network to pay attention to useful textures and ignore unimportant details.

In our design, the two pointwise convolutions in FasterBlock are substituted by the CGLU, yielding the Faster–CGLU (FC) block (Figure 4C). In this way, PConv extracts multi-scale features with a small computational cost, and CGLU adaptively reweights features by their importance. The proposed C3k2_Faster_CGLU module consists of efficient local feature extraction and adaptive global reasoning, which allows the detector to obtain more accurate recognition of maize plants and achieve solid performance improvements in complex field backgrounds.

##### Detect_LSH Module

**Figure 5:**
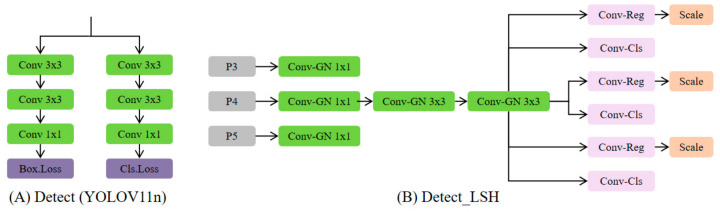
Structure diagram of detection head.

In order to compress the detection head without incurring accuracy degradation, a lightweight shared head (LSH) was proposed by optimizing the YOLOv11 detection head. The architecture of the proposed module is shown in Figure 5.

Based on shared convolutions and adaptive feature scaling, LSH has a good trade-off between model efficiency and prediction accuracy. In contrast to the typical detection head (Figure 5A), where convolving operations are done separately at each scale, the LSH (Figure 5B) is a single unified shared convolution approach. The feature maps of three scales (P3, P4 and P5) generated by the Neck are initially sent to a 1 × 1 convolution for channel alignment and normalization, so that they can have consistent channel numbers and comparable scaled magnitudes in depth. The normalized maps are then jointly fed into two shared 3 × 3 convolution layers, which share parameters across scales. This architecture avoids redundant computation and parameters, and promotes semantic consistency and more robust information flow across features at different resolutions.

After feature extraction, the shared features are separated into two prediction branches: a regression branch that predicts the bounding-box coordinates and a classification branch that predicts class confidence. At the end of each branch, a scale layer is inserted to stabilize the multi-scale predictions. This layer includes trainable coefficients that modulate feature amplitudes to account for the disparity in spatial resolutions and receptive fields among scales, providing better capabilities for the model to tackle large appearance variation. We use Group Normalization (GN) instead of Batch Normalization for better numerical stability and generalization, especially when the batch size is small, during convolution and normalization.

In summary, the LSH preserves the advantage of having a decoupled detection head—individual optimization for classification and regression—while diminishing redundancy by sharing parameters. Exploiting shared convolution and adaptive scaling, the presented Detect–LSH module considerably streamlines the detection head and reduces computation and memory requirements with a slight degradation in accuracy. Consequently, it is advantageous for real-time agricultural object detection on embedded systems with constrained processing power.

##### LAMP Pruning Strategy

In order to further reduce the computational cost of the model and to make it more mobile-friendly, we applied the Layer-Adaptive Magnitude-Based Pruning (LAMP) scheme [44] to the improved maize detection network.

LAMP learns to prune in a layer-wise saliency-aware manner, rather than directly removing the weights based on their magnitudes. LAMP evaluates the saliency of each weight and gradually removes the least salient ones. The network is tenser and faster after removing redundant parameters, but with nearly the same detection accuracy, which is also a very important feature for real-time inference on embedded hardware. The motivation for LAMP is to measure how much each weight affects the output of its layer, and exploit this measure as a criterion for pruning. Depending on the selected pruning protocol, weights with smaller importance scores are pruned either gradually (progressive pruning) or all at once (one-shot pruning). The sparsity fraction during gradual pruning increases incrementally, one layer at a time. After pruning, the model is fine-tuned to regain any potential loss in performance and to stabilize the sparse structure.

By way of this fine-grained, adaptive sparsification method, LAMP achieves a better trade-off between compression and accuracy. It greatly decreases computational and storage needs while retaining the predictive ability of the fully connected baseline model. The relative importance of every weight is given by the following quality function:

(3) $$\mathrm{score}\left(u;\;W\right)\;=\;\frac{{\left(W[u]\right)}^{2}}{\sum_{v\;\geq \;u}{\left(W[v]\right)}^{2}}$$

where score(u; W) denotes the pruning score, and W[u] and W[v] represent the weights corresponding to the sorted indices u and v, respectively. Specifically, all weights in each layer are first flattened and sorted in descending order according to their absolute values. Thus, v ≥ u indicates the index order in the sorted weight sequence, rather than the original channel index. The denominator is computed as the cumulative sum of squared weights from the current sorted index u to the end of the sequence, which is used to normalize the squared magnitude of W[u]. Weights with lower normalized scores are regarded as less important and are preferentially removed during pruning.

Instead of eliminating individual weights at random, LAMP uses a structured pruning method that also prunes whole convolutional channels or neuron connections with the least contribution. Such designs can preserve tensor continuity and regular memory access, which is friendly to hardware-level parallelization. As a result, both the computation and memory requirements are dramatically reduced at inference time, while the model architecture remains clean and easy to deploy.

The model is then fine-tuned to recover any accuracy loss caused by the parameter removal. This step of retraining is supposed to make the performance more stable and enable the network to adapt again to its new sparse form. In the end, the pruned detector achieves nearly the same precision of detection as its original model, yet operates more efficiently with significantly less computational power [45].

This layer-adaptive pruning strategy, with the help of the block-structured design, preserves the most discriminative layers while pruning computations that contribute less to the final prediction. This selective pruning leads to higher deployment efficiency and to better hardware adaptability, and hence, the approach is suitable for real-time agrotechnology and other mobile vision applications.

#### 2.2.3. Model Performance Evaluation Indices

The performance of the model was evaluated by three common evaluation metrics: precision (P), recall (R) and mean average precision (mAP). Precision represents the percentage of true positive samples among all samples of positive prediction, which also reflects the ability of the model to reduce false alarms. Recall represents the percentage of true positive samples that have been truly detected, indicating the sensitivity of the model to real targets. By averaging the AP scores across all object categories, mAP produces a single number that characterizes the overall precision vs. recall performance of a detector on that particular dataset. It captures precision/recall trade-offs at all dataset levels. Formulation of these metrics in mathematical terms is given by Equations (4)–(7).

(4) $$P=\frac{\mathrm{TP}}{\mathrm{TP}+\mathrm{FP}}$$

(5) $$R=\frac{\mathrm{TP}}{\mathrm{TP}+\mathrm{FN}}$$

(6) $$\mathrm{AP}=\int_{0}^{1}P(R)\mathrm{dR}$$

(7) $$\mathrm{mAP}=\frac{1}{n}\sum_{i=0}^{n}AP_{i}$$

In addition to the typical detection metrics, there are indicators for computing efficiency and the possible deployment of the proposed model, namely GFLOPs, Parameters, model size, and FPS-CPU. GFLOPs represent how many floating-point operations are required to perform one forward pass of the model and can be seen as a rough indicator of how much computation is involved in the model. Decreased GFLOPs mean less computational cost and faster prediction, which is recommended in real-time agricultural applications. Model size is the amount of storage required by the trained weights. It is a useful quantity for assessing how easily a model may be deployed on a device with a limited amount of memory. FPS-CPU is the number of frames the model can process per second on the CPU alone. Since many agricultural control units do not support GPU acceleration, inference speed on the CPU provides a practical perspective on the performance of the detector in the field. Therefore, this is a useful metric for evaluating system responsiveness in real time and, more importantly, for assessing the possibility of economically implementing it in real-world agricultural machinery.

### 2.3. Crop Row Line Fitting and Verification

#### 2.3.1. Linear Fitting of Crop Rows

Based on the detection results of Maize–YOLO, the location of each maize plant was calculated by the coordinates of the center point of its bounding box [46]. These centroids ultimately served to characterize the overall spatial arrangement of the plants, which essentially represent the crop row clustering.

For the detection of crop rows, there are classical methods, such as the Hough transform [47] and the least squares method (LSM) [48], for line fitting when rows are straight and aligned. These strategies are sufficient in ideal conditions where the rows of crops are almost parallel with minimal geometric deformations. In real images of the field, rows often converge toward the upper part of the frame. When image coordinate space is used for fitting, such distortions may result in significant errors, where, in some cases, the row detection process may fail. Clustering directly in the image plane also tends to be computationally intensive and less stable, particularly as the number of detected plants increases, thus slowing down the overall process [49].

These effects were compensated for by applying an Inverse Perspective Mapping (IPM) transformation before clustering and line fitting. For every image, four points were selected to map the original image coordinate system to a requested, parallelized ground plane coordinate system. This mapping defines the perspective transformation matrix M, which can be represented as:

(8) $$M\;=\;\mathrm{get}\;\mathrm{IPM}\;(P_{\mathrm{src}},\;P_{\mathrm{dst}})$$

where P_src_ denotes the coordinates of the four corner points in the original image, and P_dst_ represents the corresponding points in the target parallel coordinate system. Each plant center point is expressed in homogeneous coordinates as follows:

(9) $$P_{i}\;=\;{[x_{i},\;y_{i},\;1]}^{T}$$

The inverse perspective coordinates are then obtained through the matrix transformation as follows:

(10) $$P_{i}^{‘}\;=\;\frac{Mp_{i}}{{(Mp_{i})}_{3}}$$

where ${\left(\cdot \right)}_{3}$ represents the third component of the homogeneous coordinate, which is used for normalization. This transformation effectively eliminates the horizontal convergence effect of crop rows in the original image, resulting in a more uniform row spacing along the horizontal axis. Consequently, it simplifies the subsequent clustering process and improves the stability of row structure extraction.

In the inverse perspective coordinate system, the DBSCAN (Density-Based Spatial Clustering of Applications with Noise) algorithm was applied to the horizontal coordinates $x_{i}^{‘}$ of the plant center points to cluster them into distinct crop rows. For each point $x_{i}^{‘}$, its neighborhood is defined as follows:

(11) $$N_{\varepsilon }(x_{i}^{‘})\;=\;\left\{x_{j}^{‘}|\left|x_{j}^{‘}-x_{i}^{‘}\right|\leq \varepsilon \right\}$$

If the number of points within the neighborhood of $\left|N_{\varepsilon }(x_{i}^{‘})\right|$ is greater than or equal to the minimum sample number, the point is regarded as a core point and assigned to cluster k; otherwise, it is labeled as a noise point.

Once DBSCAN clustering was completed, each cluster was treated as a candidate crop row. To describe the geometry of these rows more precisely, the least squares method (LSM) was used to fit a line through the center points belonging to each cluster. Each cluster obtained after the inverse perspective transformation contains n points with coordinates $\left(x_{i}^{‘},y_{i}^{‘}\right)$, where $x_{i}^{‘}$ denotes the horizontal coordinate of the plant center point and $y_{i}^{‘}$ represents the vertical coordinate. Since crop rows in the image predominantly extend along the vertical (*y*) direction, $y_{i}^{‘}$ is selected as the independent variable, and $x_{i}^{‘}$ as the dependent variable in the fitting process. Accordingly, the linear model is formulated based on the least squares criterion as follows:

(12) $$x_{i}^{\prime }\;=\;my_{i}^{\prime }+c$$

where m represents the slope of the crop row, and c denotes the intercept.

After the line-fitting stage, each crop row can be expressed as a linear function in the inverse-perspective coordinate plane. For visualization and navigation analysis, the endpoints of these fitted lines are then projected back into the original image space using the inverse transformation matrix M^−1^. This back-projection allows the detected rows to be displayed directly on the input image, making their spatial layout easier to interpret.

The fitting and mapping process also helps to smooth random errors introduced by detection noise or irregular spacing among plant points. As a result, the extracted crop rows appear more continuous and stable, which is essential for reliable navigation path generation and spatial structure analysis in agricultural machinery systems.

#### 2.3.2. Evaluation of Crop Row Extraction Accuracy

Field images captured by the camera often contain several parallel crop rows. In practical navigation and path-planning scenarios, however, it is unnecessary to consider every detected row [50,51]. After detection and fitting, this study focused on the two centerlines nearest to the image midpoint, which were used as the reference rows for navigation path generation. These lines represent the rows most relevant to the vehicle’s operational area and are sufficient for stable guidance.

To assess the accuracy of the extracted crop row lines, manually annotated ground-truth centerlines were used as benchmarks. The deviation between an extracted line and its corresponding reference line was quantified by calculating their angular difference. If the slope of the ground-truth line is denoted by m_1_ and that of the detected line by m_2_, the angular error θ between the two lines can be defined as:

(13) $$\theta \;=\;\arctan\left(\frac{\left|m_{1}-m_{2}\right|}{1+m_{1}m_{2}}\right)$$

When the angle error exceeds 5° [48], the crop row extraction is considered invalid, indicating that the algorithm failed to accurately identify the corresponding row orientation.

Furthermore, to fully assess the general performance of the algorithm, the fitting time of each crop row centerline was also recorded to examine the computational efficiency. The accuracy and real-time performance of the proposed method can also be well described when the angle error and fitting time across different images are compared. These results indicate that the proposed crop row extraction method is robust and feasible in real field environments.

### 2.4. Test Platform

The hardware and software environments used for model training and testing are summarized in Table 1.

It should be noted that the GPU environment listed in Table 1 was mainly used for model training, validation, and hyperparameter tuning. In contrast, the inference speed evaluation in this study was primarily conducted on the CPU. This design was adopted because the proposed method is intended for real-time deployment on agricultural machinery, where onboard computing units are often constrained by cost, power consumption, and hardware size, and may not be equipped with high-performance GPUs. Therefore, CPU-based FPS was selected as the main indicator for evaluating the practical deployment efficiency of the proposed model. The CUDA and cuDNN configurations are reported to ensure the reproducibility of the training environment, rather than to indicate that GPU inference was used as the primary benchmark.

## 3. Results

### 3.1. Performance of YOLOv11n

A total of 1555 maize field images were collected and divided into training, validation and testing sets, with the YOLOv11n model used as the baseline. To balance model convergence speed and detection accuracy during training, the hyperparameters are fine-tuned as follows: the initial learning rate is 0.01 and the optimizer is Stochastic Gradient Descent (SGD) [48] with a momentum of 0.937. A weight decay rate of 0.0005 was used for overfitting prevention.

Mosaic augmentation and horizontal flipping were used with data augmentation, and RandAugment was applied to introduce random perturbations in color space and brightness to improve model robustness and generalization. Num_workers was set to 4, and in order to avoid overfitting, an early stopping scheme with a patience of 100 was employed. The batch size was set to 16 and the number of training epochs to 300. For fair comparison, all models are trained with the same settings.

**Figure 6:**
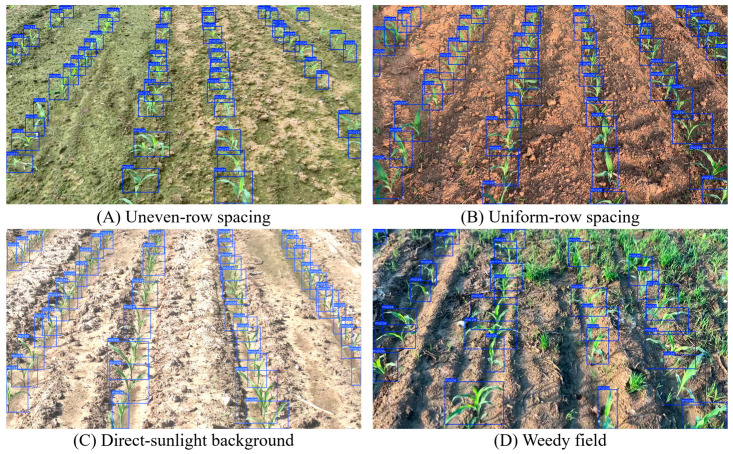
Visualization of maize plant detection results using the baseline YOLOv11n model under different field conditions.

To further illustrate the detection performance of the baseline (Table 2), some typical detection results in different field environments are shown in Figure 6. The accuracy of the model is verified across various lighting and background situations to detect maize plants accurately, and it is also robust to occlusion and background clutter. It can be seen that YOLOv11n can precisely identify individual plants, which ensures the effectiveness of feature extraction for the subsequent lightweight design and pruning analysis.

The baseline model performs well in detection across various agricultural scenes, providing a good base for the lightweight enhancement and pruning experiments shown in the following sections.

### 3.2. Performance of the Model Improved with the C3k2_Faster_CGLU Module

To evaluate the impact of the C3k2_Faster_CGLU module on detection performance, the proposed model, denoted as YOLOv11n-FC, was trained under the same hyperparameter settings, training iterations, and training strategy as the baseline. This ensured that any observed performance difference was solely attributable to the introduced architectural modification. The experimental results are presented in Table 3.

In contrast, the YOLOv11n-FC model ensured stable overall detection performance with less computational cost and storage overhead. Particularly, the GFLOPs reduced from 6.3 to 5.6, and the model size was compressed from 5.2 MB to 4.6 MB, which means the network is more compact and efficient. The FPS of CPU inference is almost unchanged, indicating that the execution speed at the terminal system is not influenced by the model with reduced computational complexity.

The improved model’s recall and mAP0.5 were almost the same, while precision was slightly worse (92.7% vs. 93.4%) than the baseline model. This shows that the C3k2_Faster_CGLU module can greatly reduce calculations with negligible accuracy sacrifice. In general, the module can achieve a desired balance between detection accuracy and computational cost, which provides an enlightening structure refinement route for further lightweight models and applications in resource-limited scenarios.

### 3.3. Performance of the Model Improved with the Detect_LSH Module

After replacing the C3k2 module with C3k2_Faster_CGLU in the YOLOv11n-FC model, the storage complexity of the model was greatly decreased; however, a small drop in precision was observed. To offset this accuracy loss in a lightweight model and with more powerful feature representation in the detection head, we proposed a Lightweight Shared Detection Head (Detect_LSH) module on the detection head of the architecture of YOLOv11n-FC, namely YOLOv11n-FCL (Maize–YOLO).

The Detect_LSH is a shared convolutional architecture that allows sharing parameters over multi-scale feature maps, including an adaptive feature-scaling mechanism to boost semantic consistency and representational ability among different feature levels. This design significantly reduces parameter redundancy and computational cost in the detection head and refines multi-scale feature fusion quality, which can improve detection accuracy while keeping computational cost unchanged.

To confirm this enhancement, we train and test the YOLOv11n-FCL with the same configuration of datasets and hyperparameters as the baseline and YOLOv11n-FC models. The detection performance is given in Table 4.

As can be seen in Table 4, the YOLOv11n-FCL model brings about a further reduction of resource consumption and achieves better detection performance. In particular, GFLOPs dropped from 5.6 to 4.9, and the model size shrank from 4.6 MB to 4.2 MB. At the same time, precision rose from 92.7% to 93.1%, and other accuracy indicators were more or less the same as those of the baseline model.

Two important inventions are introduced to improve the detection performance of Detect_LSH: (1) To begin with, the common convolutional organization allows the parameters to be shared all the way across multi-scale feature maps, so that high-level semantic information can be shared among different scales efficiently. Next, an adaptive feature-scaling approach is proposed by adapting the weighting of features based on their importance at various scales, aiming to improve the model performance toward target regions. The combined effect of both techniques is that the detection head can enjoy richer feature representation and also compensates for the small accuracy drop brought by the C3k2_Faster_CGLU module on weak or fine-grained texture targets.

Moreover, by sharing parameters, Detect_LSH also effectively avoids the repetition of calculations in the detection head and makes the deduction process more efficient. The CPU-based FPS increased to 62.1, yielding a 7.8% improvement from YOLOv11n-FC (57.6 FPS), showing better computational efficiency and real-time application potential. In summary, the addition of the Detect_LSH module benefits the overall detection model in several ways, including feature representation enhancement, detection accuracy recovery, computational efficiency improvement, and a more lightweight structural design, resulting in further advancement of the overall detection capability of the model in complicated field environments.

### 3.4. Effect of Different Pruning Ratios on Model Detection Performance

The LAMP pruning scheme was employed on the enhanced YOLOv11n-FCL model to analyze the connection between model lightweighting and detection accuracy. Different pruning ratios structurally sparsify convolutional channels, which can achieve a reduction in computational cost and parameter scale while maintaining the stability of the model backbone architecture.

All pruned models were trained and tested on the same dataset with the same set of hyperparameters for consistency and comparability during experimentation. The results of the detection accuracy of the models with different pruning ratios are given in Table 5.

As can be seen from Table 5, as the pruning ratio increased, the model parameters and GFLOPs also showed an obvious decreasing trend. The model size was compressed, and the FPS-CPU substantially increased, which demonstrated that LAMP-based structured pruning can significantly enhance computational efficiency and real-time performance without sacrificing the stability of the backbone architecture. Nevertheless, a too-high pruning rate deteriorates the feature representation power of the model and leads to poor detection accuracy and recall performance.

**Figure 7:**
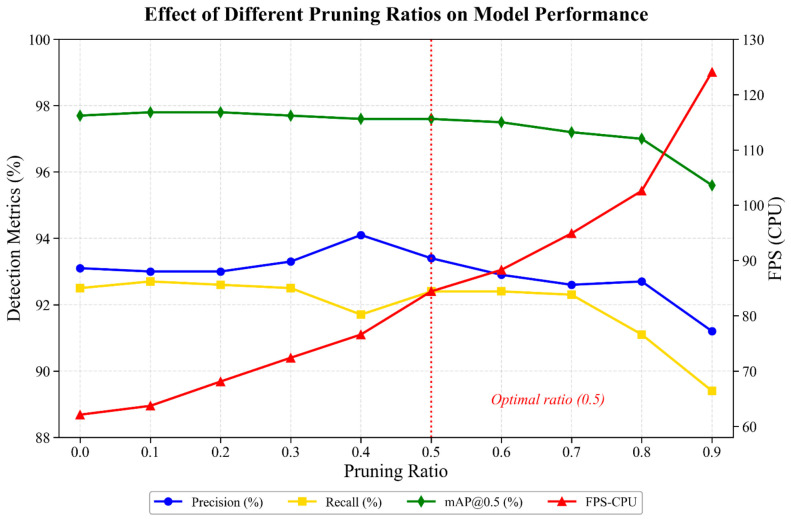
Variation in detection accuracy and computational efficiency under different pruning ratios.

**Figure 8:**
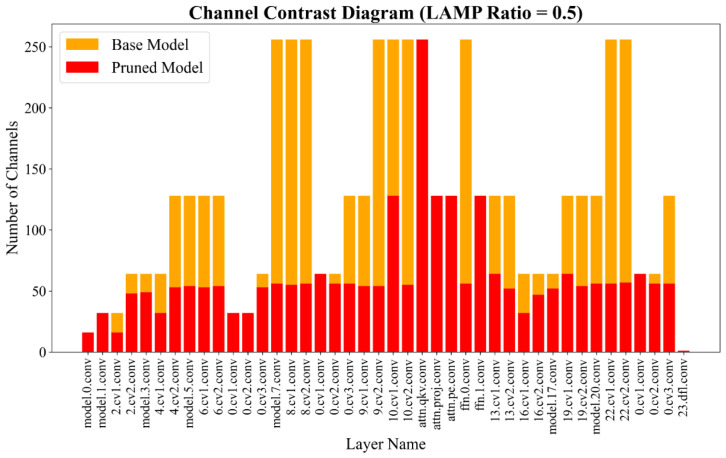
Comparison of convolutional channel numbers.

From the trends of performance (Figure 7), when the pruning rate was increased from 0 to 0.3, precision and mAP0.5 remained almost the same, with variation < 0.2%. In addition, GFLOPs dropped from 4.9 to 3.4, which meant 30.6% fewer calculations, and the FPS-CPU increased to 72.4. This indicates that mild pruning can significantly reduce computation and improve real-time performance. With the pruning rate further increased to 0.4 and 0.5, the model still exhibited strong detection performance. When a pruning ratio of 0.5 was adopted, the model obtained P 93.4%, R 92.4% and mAP0.5 97.6%, which indicates almost no accuracy loss compared with the unpruned model. A channel-wise representation of the unpruned and LAMP-pruned (0.5 rate) YOLOv11n-FCL is depicted in Figure 8. It is apparent that the LAMP technique effectively prunes redundant filters while preserving the network structure as a whole. When GFLOPs were reduced by about 51%, the model size was shrunk by about 69%, and the FPS-CPU was increased by 36% (62.1 to 84.4), the maximum balance between detection accuracy and computational cost was obtained.

When the pruning rate was higher than 0.6, GFLOPs and model storage continued to reduce, but the detection results began to degrade. Precision and recall decreased significantly; in particular, recall degraded to less than 91.1% when the rate was 0.8, which means that over-pruning causes a loss of critical information for feature extraction, leading to an inability to identify dense or occluded maize plants in complex field environments. Although the FPS-CPU is more than 100, the drastic accuracy degradation makes it inapplicable in practice.

LAMP pruning exhibits a clear nonlinear trade-off across various levels of sparsification. The model obtains mAP0.5 ≥ 97.6% with about 1.3-fold inference acceleration at pruning rates of 0.5–0.7. But further pruning will introduce more serious performance degradation. Taking accuracy, complexity and real-time factors into account, the model pruned with a 0.5 pruning ratio has the best trade-off between detection performance and lightweight efficiency, which reveals that it is possible to realize lightweighting by the proposed method for the embedded agricultural machinery platform for efficient and stable operation.

### 3.5. Ablation Experiment

To further summarize the contribution of each improvement module, a progressive ablation experiment was conducted based on YOLOv11n. The C3k2_Faster module, CGLU, Lightweight Shared Detection Head (LSH), and LAMP pruning strategy were sequentially introduced under the same dataset split, training strategy, and hyperparameter settings. The results are shown in Table 6.

As shown in Table 6, the introduction of C3k2_Faster reduced the computational cost while maintaining the same mAP@0.5 as the baseline model. The further integration of CGLU slightly reduced GFLOPs and improved CPU-based inference speed, indicating that gated feature interaction helped improve feature representation efficiency. After introducing LSH, the model achieved the highest mAP@0.5 of 97.7%, while GFLOPs decreased to 4.9 and CPU-FPS increased to 62.1. Finally, after applying LAMP pruning with a pruning ratio of 0.5, the model maintained an mAP@0.5 of 97.6%, while GFLOPs were reduced to 2.4 and CPU-FPS increased to 84.4. These results confirm that the proposed modules are complementary and jointly contribute to a lightweight, accurate, and real-time maize detection model.

### 3.6. Comparison with Other Algorithms

Under an identical training environment and dataset, the enhanced model was compared with several popular object detection algorithms, including single-stage detectors (YOLOv3–tiny, YOLOv5n, YOLOv6n, YOLOv8n, YOLOv10n, YOLOv12n and SSD) and the two-stage detector Faster R-CNN. All input parameters in the comparative experiment were strictly controlled to guarantee fairness with the same datasets and training steps. The results of the comparison are shown in Table 7.

The comparison in Table 7 was used not only to evaluate the final proposed model, but also to support the selection of YOLOv11n as the baseline for subsequent lightweight optimization. As shown in Table 7, the baseline YOLOv11n model achieved an mAP@0.5 of 97.6%, with 6.3 GFLOPs, a model size of 5.2 MB, and a CPU-based inference speed of 57.3 FPS. Compared with YOLOv3–tiny, YOLOv11n improved mAP@0.5 by 0.7 percentage points and increased CPU inference speed by approximately 50.4%, indicating that YOLOv11n provides a more favorable balance between detection accuracy and inference efficiency. Compared with other lightweight YOLO variants, including YOLOv5n, YOLOv6n, YOLOv8n, YOLOv10n, and YOLOv12n, YOLOv11n achieved comparable detection accuracy while maintaining relatively low computational complexity and stable CPU inference performance. These results support the selection of YOLOv11n as the baseline model for further lightweight optimization.

The proposed YOLOv11n-FCL (Pruned = 0.5) further improved the deployment-oriented efficiency of the baseline model. Compared with YOLOv11n, the proposed model maintained the same mAP@0.5 of 97.6%, while reducing GFLOPs from 6.3 to 2.4 and decreasing the model size from 5.2 MB to 1.3 MB. Meanwhile, the CPU-based inference speed increased from 57.3 FPS to 84.4 FPS, corresponding to an improvement of approximately 47.3%. These results indicate that the introduced C3k2_Faster_CGLU module, Detect_LSH module, and LAMP pruning strategy effectively reduce redundant computation and model storage while preserving detection accuracy.

It should be noted that the superiority of the proposed model is mainly reflected in its overall accuracy–efficiency trade-off rather than in a large absolute improvement in detection accuracy alone. For example, YOLOv12n achieved a slightly higher mAP@0.5 of 97.7%, but required 5.8 GFLOPs and 5.2 MB of storage, and its CPU-based inference speed was only 48.4 FPS. In contrast, the proposed model achieved a comparable mAP@0.5 of 97.6%, while reducing GFLOPs by 58.6%, decreasing model size by 75.0%, and improving CPU inference speed by 74.4% compared with YOLOv12n. This demonstrates that the proposed model is more suitable for resource-constrained agricultural deployment scenarios.

Compared with traditional detectors, the proposed model also showed clear advantages. SSD obtained an mAP@0.5 of 87.4% and a CPU inference speed of 12.8 FPS, while Faster R-CNN achieved an mAP@0.5 of 93.2% but only 1.21 FPS. In comparison, the proposed YOLOv11n-FCL (Pruned = 0.5) achieved higher detection accuracy and much faster CPU inference speed. Specifically, its CPU inference speed was approximately 6.6 times that of SSD and about 69.8 times that of Faster R-CNN, while its computational cost was only about 7.9% and 0.51% of SSD and Faster R-CNN, respectively.

In summary, although several lightweight YOLO models achieved similar detection accuracy, the proposed Maize–YOLO model achieved the best overall balance among mAP@0.5, GFLOPs, model size, and CPU-based inference speed. Therefore, the proposed YOLOv11n-FCL (Pruned = 0.5) can provide an effective and practical lightweight solution for real-time maize plant detection and embedded deployment in autonomous agricultural navigation.

### 3.7. Detection of Crop Row Centerlines

The predicted outputs of the model also contain the x and y coordinates of the center location of each predicted bounding box, which can be directly used as position information for an individual maize plant. Because of the perspective projection in camera imaging, those nearest parallel crop rows captured by the camera in the field do not run exactly parallel; they converge from the bottom of an image to the top of the image. Thus, if the x and y values of the kernel are used for clustering simultaneously, kernels in one cluster are no longer guaranteed to belong to a single row. This decreases not only the precision of row detection and navigation path generation but also the computational cost of the clustering process, which is a real-time system-degrading factor [49].

To overcome this problem, in the detected images, the Inverse Perspective Mapping (IPM) is applied first, which converts the original image with the camera’s perspective view to an orthographic top-view image without camera calibration. This process compensates for perspective distortion, resulting in the rows being approximately parallel in the IPM images. As a consequence, it can guarantee higher precision and better real-time performance for crop row detection by alleviating the challenges of subsequent clustering processes.

**Figure 9:**
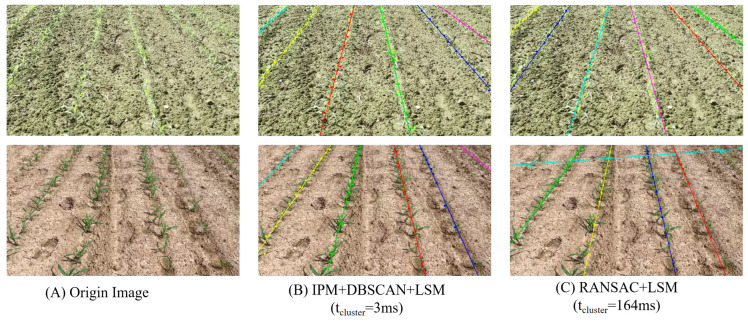
Crop row detection results under different clustering methods.

To further validate the effectiveness of the proposed IPM preprocessing, a comparative experiment was performed between the traditional RANSAC + LSM approach and the IPM + DBSCAN + LSM pipeline. As shown in Figure 9, the proposed method significantly reduced the clustering and line-fitting time from 164 ms to 3 ms, representing a huge improvement in computational efficiency. This result confirms that the IPM transformation effectively alleviates perspective distortion, reduces redundant spatial computation during clustering, and greatly enhances the real-time performance of the crop row detection process.

After the IPM transformation and based on the results obtained—where the crop rows are mainly aligned along the vertical direction—only the x-coordinates of the centers of the plants were considered in the clustering analysis within the rows. Such an approach eliminates redundant dimensions and allows more efficient computations. In addition, since a plant at the image border rarely establishes a full row, and hence the number of plants detected in a row can be used as an indicator of whether a row is complete or not, a simple threshold-based outlier removal procedure was run as a pre-processing step to clustering to get rid of outliers in each cluster.

**Figure 10:**
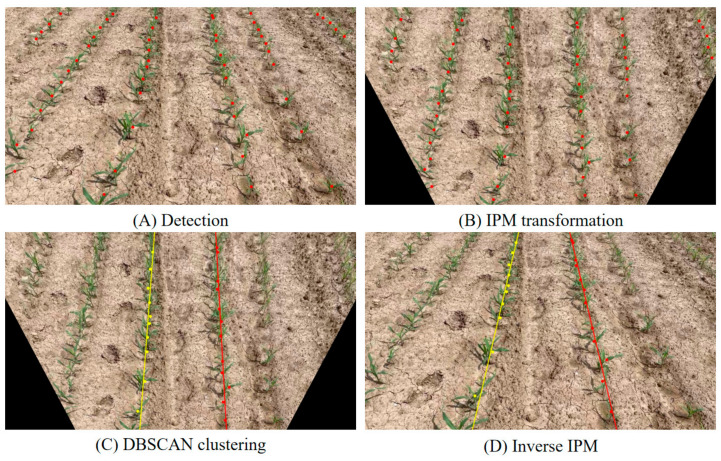
Stepwise visualization of maize row detection and navigation line extraction. (**A**) Detection results obtained using the proposed Maize–YOLO network. (**B**) Perspective transformation (IPM) to normalize crop row spacing. (**C**) DBSCAN clustering and line fitting in the IPM domain. (**D**) Visualization of the final fitted centerlines after inverse IPM transformation in the original camera view.

In practical navigation scenarios, while the agricultural machinery travels along crop row bands, not all crop rows detected in an image are needed. Hence, in this work, to maintain a trade-off between representativeness and computational burden, only the two crop row centerlines nearest to the image center were designated as the target navigation lines, as shown in Figure 10.

Given the clustering results achieved from the DBSCAN algorithm, the crop row centerlines were fitted using three different fitting approaches: Principal Component Analysis (PCA), Random Sample Consensus (RANSAC), and the least squares method (LSM). The performance of the methods was analyzed and compared with respect to three quantitative measures: time for fitting (t), angular deviation (θ), and the accuracy of the centerline detection (P).

The processing time is the total processing time of the Maize–YOLO model on the CPU that includes detection and recognition of the raw input image, which reveals the computational efficiency of each fitting method and their accuracy in a real-field navigation application. The corresponding evaluation metrics are defined as follows:

(14) $$t\;=\;\frac{1}{N}\sum_{i\;=\;1}^{N}t_{i},\mathrm{FPS}\;=\;\frac{1}{t}$$

(15) $$\theta =\frac{1}{N}\sum_{i=1}^{N}\left(\frac{1}{q}\sum_{j=1}^{q}\arctan\left|\frac{k_{1j}-k_{2j}}{1+k_{1j}k_{2j}}\right|\right)$$

(16) $$\delta =\frac{1}{N}\sum_{i=1}^{N}\frac{1}{q}\sum_{j=1}^{q}\left|x_{1j}-x_{2j}\right|$$

where *j* denotes the *j*-th crop row centerline in the image, *q* is the total number of centerlines in the image, N represents the number of test images in the dataset, $t_{i}$ refers to the fitting time (ms) for the *i*-th image, and $k_{1j}$ and $k_{2j}$ represent the slopes of the ground-truth and fitted centerlines of the *j*-th crop row, respectively. $x_{1j}$ and $x_{2j}$ denote the horizontal coordinates (in pixels) of the ground-truth and fitted crop row centers at the same vertical position.

When the angular deviation between the fitted centerline and the ground-truth centerline was less than 5°, the fitting result was considered valid. Based on this criterion, crop row centerline detection accuracy (P) was defined as the ratio of valid fitted centerlines to the total number of actual crop rows. The results obtained using RANSAC, PCA, and the proposed LSM fitting method are summarized in Table 8.

Since the final objective of this study is autonomous agricultural navigation, the crop row extraction performance was evaluated not only by detection accuracy but also by navigation-oriented geometric metrics. The valid row extraction accuracy was used to evaluate whether the fitted centerlines could be successfully generated, the mean angular deviation was used to quantify row-orientation estimation error, and the mean center deviation was used to measure the lateral localization error of the extracted navigation lines. In addition, CPU-based processing speed was used to evaluate the real-time performance of the complete perception pipeline, including maize detection, IPM transformation, clustering, and line fitting.

Table 8 shows that LSM delivered the best overall performance for crop row centerline detection. Its detection accuracy reached 98.6%, matching PCA and clearly exceeding that of RANSAC (90.1%). The mean angular deviation of LSM was 0.442°, which was much lower than the 1.938° of RANSAC, indicating more stable row orientation estimation. The mean center deviation of LSM was 2.24 ± 3.08 px, identical to PCA and substantially lower than that of RANSAC (6.51 ± 36.22 px), showing that LSM also achieved high geometric accuracy in centerline localization.

LSM also showed the best real-time performance. Its average CPU-based speed reached 22.53 FPS, which was about 13.6% higher than PCA and more than 50% higher than RANSAC. This indicates that LSM is more suitable for real-time navigation tasks in field environments.

These results further demonstrate that the proposed Maize–YOLO + IPM–DBSCAN–LSM pipeline can provide stable navigation line geometry for autonomous agricultural navigation. Unlike detection-only evaluation metrics, angular deviation and centerline deviation directly reflect the reliability of row orientation and lateral position estimation, which are critical for path tracking and steering control. The proposed LSM-based pipeline achieved a valid row extraction accuracy of 98.6%, a mean angular deviation of 0.442°, and a mean center deviation of 2.24 px, while maintaining a CPU-based processing speed of 22.53 FPS. These results indicate that the proposed method satisfies both geometric accuracy and real-time requirements for vision-based crop row perception.

These differences are mainly related to the computational characteristics of the three methods. RANSAC depends on repeated random sampling and iterative model evaluation. While this improves robustness to outliers, it also increases computational cost, especially when the number of points becomes large. PCA requires covariance calculation and eigenvalue decomposition, and its computational burden increases as the data scale grows. In contrast, LSM directly fits a least squares line to the clustered plant center points using a single matrix solution. This makes the fitting process simpler and more stable, which helps improve efficiency while preserving fitting accuracy comparable to that of PCA.

Because crop rows in the transformed image approximately follow a linear distribution, LSM is able to exploit this structure effectively without the additional feature decomposition used in PCA. As a result, it provides a strong balance between accuracy and speed. Overall, LSM performed reliably under field conditions with relatively regular crop rows and moderate noise, and can provide fast and stable centerline estimation for autonomous agricultural navigation. Therefore, LSM was selected as the final fitting method for subsequent crop row centerline extraction.

## 4. Discussion

In this study, a lightweight vision-based framework, referred to as Maize–YOLO, was developed to address the practical trade-off among detection accuracy, computational cost, and real-time performance in autonomous agricultural navigation under complex field conditions. Unlike many existing methods that focus mainly on either accuracy or speed, the proposed framework places particular emphasis on deployment feasibility under resource-limited hardware conditions. This is a key requirement in practical agricultural machinery, yet it is not always adequately considered in previous studies.

In the broader context of agricultural robot navigation, visual navigation has become an important research direction for both open-field and controlled-environment applications. A recent review on visual navigation for agricultural robots summarized that vision-based navigation systems are expected to provide reliable environmental perception for tasks such as crop row following, path guidance, obstacle avoidance, and autonomous field operation [52]. However, these systems still face several practical challenges, including outdoor illumination variations, shadows, occlusions caused by leaves and weeds, irregular crop growth, and the trade-off between perception accuracy and real-time deployment efficiency. These challenges are particularly important for crop row navigation, where the perception system must not only recognize crop targets but also provide stable geometric information for path tracking and steering control. In this context, the proposed Maize–YOLO + IPM–DBSCAN–LSM framework provides a plant-level structural perception solution. Instead of relying on dense pixel-level segmentation, the method detects individual maize plants and reconstructs crop row centerlines from their spatial distribution. This design allows the system to exploit the regularity of row-planted crops while reducing computational burden, thereby improving the balance among robustness, geometric accuracy, and real-time deployment efficiency.

### 4.1. Lightweight Detection Design for Real-Field Deployment

For maize plant detection, Maize–YOLO combines three complementary design strategies: the C3k2_Faster_CGLU feature extraction module, the Lightweight Shared Detection Head (Detect_LSH), and the LAMP pruning strategy. These components work together to reduce redundant computation and compress the model while maintaining effective feature representation. The experimental results indicate that this design achieves a strong balance between detection performance and efficiency, allowing the model to retain high accuracy with a much lower computational burden. More importantly, the CPU-based inference speed obtained in this study suggests that the model is suitable for deployment on onboard processors commonly used in agricultural robots, without the need for high-performance GPU support.

Compared with other lightweight YOLO variants, the proposed detector achieves competitive accuracy while improving inference efficiency. This is particularly important for continuous navigation tasks, where delays in visual perception can directly affect control response, operational stability, and field safety.

### 4.2. Structural Reconstruction of Crop Rows from Plant-Level Perception

Beyond plant detection itself, this study treats crop row perception as a structural reconstruction problem rather than a task driven only by local visual appearance. Starting from individual plant detections, an IPM–DBSCAN–LSM pipeline was used to recover the global geometry of crop rows. The Inverse Perspective Mapping (IPM) step compensates for perspective convergence in camera images and transforms the scene into a quasi-top-view representation, where crop rows become approximately parallel. This makes the subsequent clustering and line fitting more stable.

In the IPM domain, the clustering problem is simplified to a lower-dimensional form, allowing DBSCAN to separate crop rows according to spatial density more robustly. The least squares method (LSM) then smooths local noise in the plant distribution and produces continuous centerlines with good geometric consistency. In this way, the proposed framework achieves accurate crop row extraction with low computational cost, which is important for real-time navigation tasks.

Since the final objective of this study is autonomous agricultural navigation, the performance of the complete perception pipeline should be evaluated not only by detection metrics but also by navigation-related geometric indicators. As reported in Table 8, the proposed Maize–YOLO + IPM–DBSCAN–LSM pipeline achieved a valid row extraction accuracy of 98.6%, a mean angular deviation of 0.442°, a mean center deviation of 2.24 px, and a CPU-based processing speed of 22.53 FPS. These indicators directly reflect the reliability of row-orientation estimation, lateral centerline localization, and real-time navigation line generation.

To further evaluate the effectiveness of this plant-level structural reconstruction strategy, Table 9 compares the processing efficiency of the proposed method with several representative segmentation-based and detection-based crop row detection or navigation line extraction approaches. It should be noted that the reported processing speeds were collected from different studies and may vary depending on crop type, dataset, hardware configuration, implementation details, and whether the reported value refers to total pipeline time or recognition time only. Moreover, many existing studies do not simultaneously report navigation-related metrics such as angular deviation, centerline deviation, valid row extraction rate, and total pipeline latency. Therefore, the comparison in Table 9 mainly provides a practical reference for processing efficiency and deployment potential rather than a strictly uniform benchmark across all metrics.

Table 9 shows that the proposed framework offers faster overall processing than several representative crop row detection and navigation line extraction methods, while maintaining strong geometric accuracy, as shown in Table 8. Compared with segmentation-based methods such as U-Net-based and E-Net-based approaches, the proposed method avoids dense pixel-level prediction and reconstructs row geometry from plant-level detections, which reduces computational burden. Compared with other detection-based methods, the proposed pipeline achieves a competitive or higher processing speed while providing explicit navigation line geometry through IPM transformation, DBSCAN clustering, and LSM fitting. These results suggest that reconstructing crop rows from plant-level detections is a practical alternative to dense segmentation, especially when both real-time performance and deployment efficiency are required for autonomous agricultural navigation.

### 4.3. From Pixel-Level Segmentation to Plant-Level Structural Perception

Traditional segmentation-based methods for crop row detection [32,58] generally depend on dense pixel-level prediction and often assume that crop rows remain visually continuous and that plant spacing is relatively uniform. In actual field conditions, however, these assumptions are often disrupted by uneven emergence, missing plants, weed interference, and changing illumination. As a result, the segmented row masks may become fragmented, which can in turn lead to unstable navigation line extraction. This reflects a broader limitation of segmentation-based methods: they focus mainly on visual continuity, which does not always align with the geometric demands of navigation.

In contrast, autonomous navigation is more directly related to geometric consistency and topological stability. For this reason, the proposed framework uses a plant-centric perception strategy, where crop rows are inferred from the spatial distribution of discrete plant centers instead of continuous pixel masks. With this formulation, crop row geometry can still be recovered even when visual cues are incomplete, as long as enough plant detections are preserved.

The use of IPM further improves this strategy by transforming plant distributions into a more physically meaningful coordinate space. This reduces ambiguity during clustering and improves robustness to perspective distortion. In this way, the detection–IPM–clustering–fitting pipeline separates visual perception from geometric reconstruction more clearly, which helps produce more reliable crop row estimates under complex field conditions.

This shift from pixel-level segmentation to plant-level structural perception provides a more robust and efficient solution for crop row detection in autonomous agricultural navigation. The present results indicate that exploiting the spatial regularity inherent in row-planted crops can improve both practical reliability and deployment efficiency, and may offer a useful direction for future vision-based navigation systems in precision agriculture.

Despite these promising results, some deployment-oriented limitations remain. First, the CPU inference tests in this study were conducted on a workstation platform rather than on embedded controllers commonly used in agricultural machinery. Therefore, further validation on edge computing boards or vehicle-mounted controllers is still required. Second, this study mainly evaluated image-level maize detection and crop row centerline extraction performance. Continuous closed-loop navigation experiments under real field operation conditions, including vehicle control response, vibration disturbance, and long-duration environmental variation, have not yet been conducted. Future work will focus on embedded deployment and real-time field navigation validation to further verify the practical applicability of the proposed framework.

In addition to deployment-related limitations, the generalization ability of the proposed method also requires further validation. Although the dataset used in this study was collected from three different locations under different weather conditions, background textures, and planting patterns, the experiments were mainly conducted on maize seedling images. Therefore, the applicability of the proposed Maize–YOLO + IPM–DBSCAN–LSM pipeline to other crop types, different growth stages, curved crop rows, missing plants, severe weed interference, and highly irregular planting patterns has not yet been fully demonstrated. The current pipeline relies on accurate plant detection and the spatial regularity of row-planted crops. When crop rows are strongly curved, plant emergence is discontinuous, weeds are visually similar to crop seedlings, or planting patterns are highly irregular, clustering and line fitting may become less stable. Future work will therefore extend the validation dataset to include different crops, multiple growth stages, and more complex field environments. In addition, temporal information from video sequences will be investigated to improve the stability of crop row tracking, and multimodal fusion with GNSS, LiDAR, or IMU data will be explored to further enhance navigation robustness under challenging field conditions.

## 5. Conclusions

This study developed a modified YOLOv11n-based framework for maize plant detection and crop row extraction to address the main limitations of traditional vision-based agricultural navigation, namely insufficient detection accuracy, limited real-time performance, and high computational cost. The detection network was progressively optimized by introducing the C3k2_Faster_CGLU feature extraction block, the Lightweight Shared Detection Head (Detect_LSH), and the LAMP pruning strategy. These improvements reduced model complexity substantially while maintaining strong detection accuracy. The final Maize–YOLO model achieved an mAP@0.5 of 97.6%, reduced GFLOPs from 6.3 to 2.4, and decreased model size by about 75%. At the same time, CPU-based inference speed increased from 57.3 to 84.4 FPS, showing that the proposed detector can achieve a strong balance between accuracy and real-time efficiency.

For crop row extraction, an integrated IPM–DBSCAN–LSM pipeline was proposed. The IPM transformation reduced perspective distortion and made the row structure more regular in the transformed image, which improved the reliability of clustering and line fitting. Using this pipeline, the method achieved a crop row centerline detection accuracy of 98.6%, with an average angular deviation of 0.442° and a CPU-based processing speed of 22.53 FPS. These results indicate that the proposed framework can provide accurate and efficient crop row perception for practical field navigation.

The proposed Maize–YOLO and IPM–clustering method provides a robust and efficient vision-based solution for autonomous agricultural navigation. Future work will focus on extending the method to different crop types and growth stages, and on incorporating multimodal sensing information, such as stereo vision and LiDAR point clouds, to support 3D crop row reconstruction and dynamic scene modeling. This may further improve the capability of agricultural robots in intelligent field operations and precision path planning.

## Figures and Tables

**Table 1:** Test platform.

Configuration	Parameter
Operating System	Windows 10 Professional Workstation Edition
CPU	Intel i7 13600KF
GPU	Nvidia RTX4070ti 12 GB
Accelerate Environment	Window 10 Professional
Python	3.8
Pytorch	2.0.1
Cuda	11.8
Cudnn	8.9.1
Data Annotation Tools	LabelImg (Version 1.8.6)

**Table 2:** Detection performance of the baseline.

Model	P/%	R/%	mAP0.5/%	GFLOPs	Model Size/MB	FPS-CPU
YOLOv11n	93.4	92.7	97.6	6.3	5.2	57.3

**Table 3:** Detection performance of YOLOv11n-FC.

Model	P/%	R/%	mAP0.5/%	GFLOPs	Model Size/MB	FPS-CPU
YOLOv11n-FC	92.7	92.6	97.5	5.6	4.6	57.6

**Table 4:** Detection performance of YOLOv11n-FCL.

Model	P/%	R/%	mAP0.5/%	GFLOPs	Model Size/MB	FPS-CPU
YOLOv11n-FCL	93.1	92.5	97.7	4.9	4.2	62.1

**Table 5:** Detection performance of YOLOv11n-FCL under different pruning ratios.

Model	Ratio	P/%	R/%	mAP0.5/%	GFLOPs	Model Size/MB	FPS-CPU
YOLO v11n-FCL	0	93.1	92.5	97.7	4.9	4.2	62.1
	0.1	93.0	92.7	97.8	4.4	3.2	63.7
	0.2	93.0	92.6	97.8	3.9	2.6	68.1
	0.3	93.3	92.5	97.7	3.4	2.0	72.4
	0.4	94.1	91.7	97.6	2.9	1.6	76.6
	0.5	93.4	92.4	97.6	2.4	1.3	84.4
	0.6	92.9	92.4	97.5	1.9	1.1	88.3
	0.7	92.6	92.3	97.2	1.4	0.9	94.9
	0.8	92.7	91.1	97.0	0.9	0.7	102.6
	0.9	91.2	89.4	95.6	0.5	0.6	124.1

**Table 6:** Ablation experiment results.

Model	C3k2_Faster	CGLU	LSH	LAMP (0.5)	mAP@0.5/%	GFLOPs	CPU-FPS
YOLOv11n	/	/	/	/	97.6	6.3	57.3
YOLOv11n-F	√	/	/	/	97.6	5.8	55.4
YOLOv11-FC	√	√	/	/	97.5	5.6	58.6
YOLOv11-FCL	√	√	√	/	97.7	4.9	62.1
YOLOv11-FCL LAMP(0.5)	√	√	√	√	97.6	2.4	84.4

**Table 7:** Comparison of detection performance among different object detection models.

Model	P/%	R/%	mAP0.5/%	GFLOPs	Model Size/MB	FPS-CPU
YOLOv11n-FCL (Pruned = 0.5)	93.4	92.4	97.6	2.4	1.3	84.4
YOLOv11n (Baseline)	93.4	92.7	97.6	6.3	5.2	57.3
YOLOv3–tiny	91.9	92.2	96.9	14.3	18.3	38.1
YOLOv5n	93.5	92.2	97.6	5.8	4.4	42.8
YOLOv6n	93.4	92.3	97.5	11.5	8.2	51.1
YOLOv8n	93.5	92.3	97.5	6.8	5.4	53.3
YOLOv10n	93.6	91.0	97.1	6.5	4.6	57.1
YOLOv12n	93.2	92.6	97.7	5.8	5.2	48.4
SSD	90.0	64.9	87.4	30.43	90.6	12.8
Faster-RCNN	77.7	94.1	93.2	470.5	108.0	1.21

**Table 8:** Statistical evaluation metrics for crop row centerline detection using different fitting methods.

Methods	P/%	Mean Angle Deviation/°	Mean Center Deviation/px	CPU-Based Speed/FPS
LSM	98.6	0.442 ± 0.799	2.24 ± 3.08	22.53
PCA	98.6	0.443 ± 0.800	2.24 ± 3.08	19.83
RANSAC	90.1	1.938 ± 2.015	6.51 ± 36.22	14.60

**Table 9:** Comparison of crop row recognition and processing speeds under different algorithms.

Algorithm	Method Type	Target Crop	Processing Speed/FPS	Reference
Maize–YOLO	Detection-based + clustering/fitting	Maize	22.53 (total)/CPU-based 41.34 (total)/GPU-based	ours
YOLOv5-M3	Detection-based	Maize	≤19.6 (total)	[53]
CAROLIF	Traditional vision-based	Maize	9.2 (total)/CPU-based	[54]
YOLOv5	Detection-based	Pineapple	17.35 (recognition)	[55]
YOLOv5-based	Detection-based	Paddy	3.6 (total)	[49]
U-Net-based	Segmentation-based	Potato	1.9 (total)	[34]
E-Net-based	Segmentation-based	Sugar beet	17 (recognition)	[56]
Hippopotamus Optimization	Optimization Optimization-based/traditional vision	Peanut, soybean, cabbage	4.2 (total)	[57]

## Data Availability

The dataset generated and analyzed in this study is not publicly available as it is part of an ongoing research project. If you wish to access the dataset, please contact the corresponding author. The author will review each request individually, taking into account legal, ethical, and scientific considerations.
